# Mitochondrial-nuclear crosstalk, haplotype and copy number variation distinct in muscle fiber type, mitochondrial respiratory and metabolic enzyme activities

**DOI:** 10.1038/s41598-017-14491-w

**Published:** 2017-10-25

**Authors:** Xuan Liu, Nares Trakooljul, Frieder Hadlich, Eduard Murani, Klaus Wimmers, Siriluck Ponsuksili

**Affiliations:** 1 Research Unit ‘Functional Genome Analysis’, Leibniz Institute for Farm Animal Biology (FBN), Wilhelm-Stahl-Allee 2, D-18196 Dummerstorf, Germany; 2 Research Unit ‘Genomics’, Leibniz Institute for Farm Animal Biology (FBN), Wilhelm-Stahl-Allee 2, D-18196 Dummerstorf, Germany

## Abstract

Genes expressed in mitochondria work in concert with those expressed in the nucleus to mediate oxidative phosphorylation (OXPHOS), a process that is relevant for muscle metabolism and meat quality. Mitochondrial genome activity can be efficiently studied and compared in Duroc and Pietrain pigs, which harbor different mitochondrial haplotypes and distinct muscle fiber types, mitochondrial respiratory activities, and fat content. Pietrain pigs homozygous-positive for malignant hyperthermia susceptibility (PiPP) carried only haplotype 8 and showed the lowest absolute mtDNA copy number accompanied by a decrease transcript abundance of mitochondrial-encoded subunits *ND1*, *ND6*, and *ATP6* and nuclear-encoded subunits *NDUFA11 and NDUFB8*. In contrast, we found that haplotype 4 of Duroc pigs had significantly higher mitochondrial DNA (mtDNA) copy numbers and an increase transcript abundance of mitochondrial-encoded subunits *ND1*, *ND6*, and *ATP6*. These results suggest that the variation in mitochondrial and nuclear genetic background among these animals has an effect on mitochondrial content and OXPHOS system subunit expression. We observed the co-expression pattern of mitochondrial and nuclear encoded OXPHOS subunits suggesting that the mitochondrial-nuclear crosstalk functionally involves in muscle metabolism. The findings provide valuable information for understanding muscle biology processes and energy metabolism, and may direct use for breeding strategies to improve meat quality and animal health.

## Introduction

Mitochondria are involved in many key cellular processes such as apoptosis, calcium homeostasis, reactive oxygen species production, and most importantly, adenosine triphosphate (ATP) generation by oxidative phosphorylation (OXPHOS). In addition, these organelles contain their own DNA distinct from the nuclear genome. In pigs, mitochondrial DNA (mtDNA) is 16,613 base pairs in length and encodes two ribosomal RNA (rRNA), 22 transfer RNA (tRNA), and 13 protein-coding genes involved in the OXPHOS system. Since OXPHOS subunits are encoded by both the nuclear and mitochondrial genome, the two genome systems interact intricately to form the fully assembled functional OXPHOS complexes.

Mitochondrial genetic variation can affect fertility, longevity, and evolutionary trajectories and acts as a human health indicator^1^. mtDNA haplotype variation affects metabolic performance in many species, such as raccoons, dogs, cows, and pigs^2^, and mitochondrial DNA (mtDNA) copy number has been associated with extensive exercise, age-related hearing impairment, disordered antioxidant capacity, and heart failure^3–6^. In *Drosophila*, mtDNA copy number is proposed to be modulated by mtDNA genome variation^7^. Mitochondrial DNA haplotypes are potential targets for manipulating phenotypes including tolerance to heat, growth, and milk quality in farm animals^8^. Hence, an understanding of the influence of mitochondrial haplotypes on energy metabolism in pigs would provide valuable information on mitochondrial function and inform farming practices to improve meat quality and animal health. Since pigs share many similarities with humans in terms of genetics and physiology, this knowledge is also potentially applicable to human diseases.

In addition to variation in mitochondrial density among muscle types, mitochondria are functionally optimized and specialized in glycolytic and oxidative fibers^9^. Mitochondria isolated from muscle immediately after slaughter are similar to those found in intact muscle, whereas some mitochondria from pale, soft, exudative (PSE) muscle are already swollen and show a decreased matrix density^10^. With an emphasis placed on glycolysis, mitochondrial content and function may be important factors contributing to postmortem muscle metabolism^11^. Duroc and Pietrain are two commercial pig breeds known for divergent meat quality and muscular energy metabolism. Muscle from Duroc pigs typically contains more slow-twitch oxidative (STO) fibers and intramuscular fat, whereas Pietrain pigs are leaner and their muscles contain more fast-twitch glycolytic (FTG) fibers^12–14^. The content of different muscle fibers types, their size and structure largely contribute to differences in growth performance and carcass traits as well as postmortem meat quality traits^15^. Lipids are stored mainly in STO fibers, which can improve the tenderness and juiciness of the meat^16^. The selection of a high percentage of FTG fibers may result in altered meat quality possibly due to the lower capillarization, insufficient delivery of oxygen and glycogen depletion^17,18^. Indeed, the meat quality parameters such as color of the meat, drip loss and shear force were measured in muscle of the same pigs^12,14^. PiPP pigs showed increased drip loss and shear force comparing to other three pig breeds. These results directly supported the association between muscle fiber type and meat quality. In Pietrain pigs, mutations within the ryanodine receptor 1 (RYR1) are associated with malignant hyperthermia susceptibility (MHS), reduced water holding capacity, and increased PSE meat^19–21^. Thus, Duroc and Pietrain pigs are unique models in which to study mitochondrial properties and energy metabolism influencing muscle metabolism and meat quality.

We have previously shown the transcriptional signatures in muscle from these pigs to be related to metabolic properties and mitochondrial respiration^22–24^. Furthermore, we wanted to know the mtDNA variation of these pigs and investigate whether mtDNA variation preferentially modifies the expression of mitochondria-associated OXPHOS gene from both mitochondria and nuclear genomes. Finally, we investigated mtDNA variation genes involved in mtDNA copy number in conjunction with muscle fiber types and metabolic enzyme activities in postmortem longissimus muscles (LM) from four different pig breeds: Duroc, Pietrain homozygous-negative for MHS (PiNN), Pietrain homozygous-positive for MHS (PiPP), and an F2 crossbred Duroc-Pietrain homozygous-negative for MHS (DuPi).

## Results

### Phenotypic differences among breeds

To illustrate breed differences in muscle metabolism, muscle fiber composition, metabolic activities, and pH were compared among the four breeds. Duroc pigs had the highest percentage of STO muscle fibers and lower fast-twitch oxidative (FTO) fibers (Supplementary Fig. S1). PiPP pigs had a significantly higher percentage of FTG fibers (80.8%) compared to PiNN pigs (74.9%, *p* = 0.02), while Duroc and DuPi were in between the two Pietrain pigs.

The activities of key metabolic enzymes for energy metabolism were measured for all four pig breeds. PiNN pigs had the highest activity of phosphofructokinase (PFK), whereas no significant differences in the activities of glycogen phosphorylase (GP) and lactate dehydrogenase (LDH) were detected among breeds. Duroc pigs had the highest complex I activity (12.5 U/g protein) compared to other breeds. Moreover, PiPP had significantly lower pH than the other three breeds.

All measured phenotypic traits were compared at time 0 and 30 min postmortem (Supplementary Fig. S2). Most of the phenotypes, except complex IV activity, were significantly different between time points. The enzyme activities of PFK and LDH were increased from 475 to 859 U/g protein (*p* = 0.0002) and from 10.9 to 15 U/g protein (*p* < 0.0001), respectively, whereas the oxidative enzyme activities of CS, complex I and complex II, together with GP activity and pH, were decreased at 30 min postmortem compared to immediately after slaughter (*p* values ranging from < 0.0001 to 0.02).

### Different mitochondrial haplotypes among pig breeds

The D-loop regions in 53 animals were sequenced and eight haplotypes were identified. For statistical reasons, five haplotypes identified in at least three animals were included in subsequent data analysis. Detailed haplotype information is shown in Supplementary Table 1 and Fig. S3. In brief, haplotypes 4 (Duroc: 10, DuPi: 6) and 6 (Duroc: 5, DuPi: 1) were present in mainly Duroc and DuPi pigs whereas haplotype 1 was present in five DuPi pigs only. Haplotype 7 was present in three PiNN pigs, while haplotype 8 was found in 14 PiPP and four PiNN pigs. Muscles from pigs with haplotype 7 contained significantly more FTO muscle fibers than haplotypes 4, 6, and 8 (*p* < 0.05, Fig. S4). Interestingly, haplotype 8 showed the lowest complex I activity among all the haplotypes and had significantly lower activity than haplotypes 4 and 6 (*p* < 0.05). All other phenotypic traits were comparable between haplotypes.

### Duplication of the porcine mitochondrial genome in the nuclear genome

**Figure 1:**
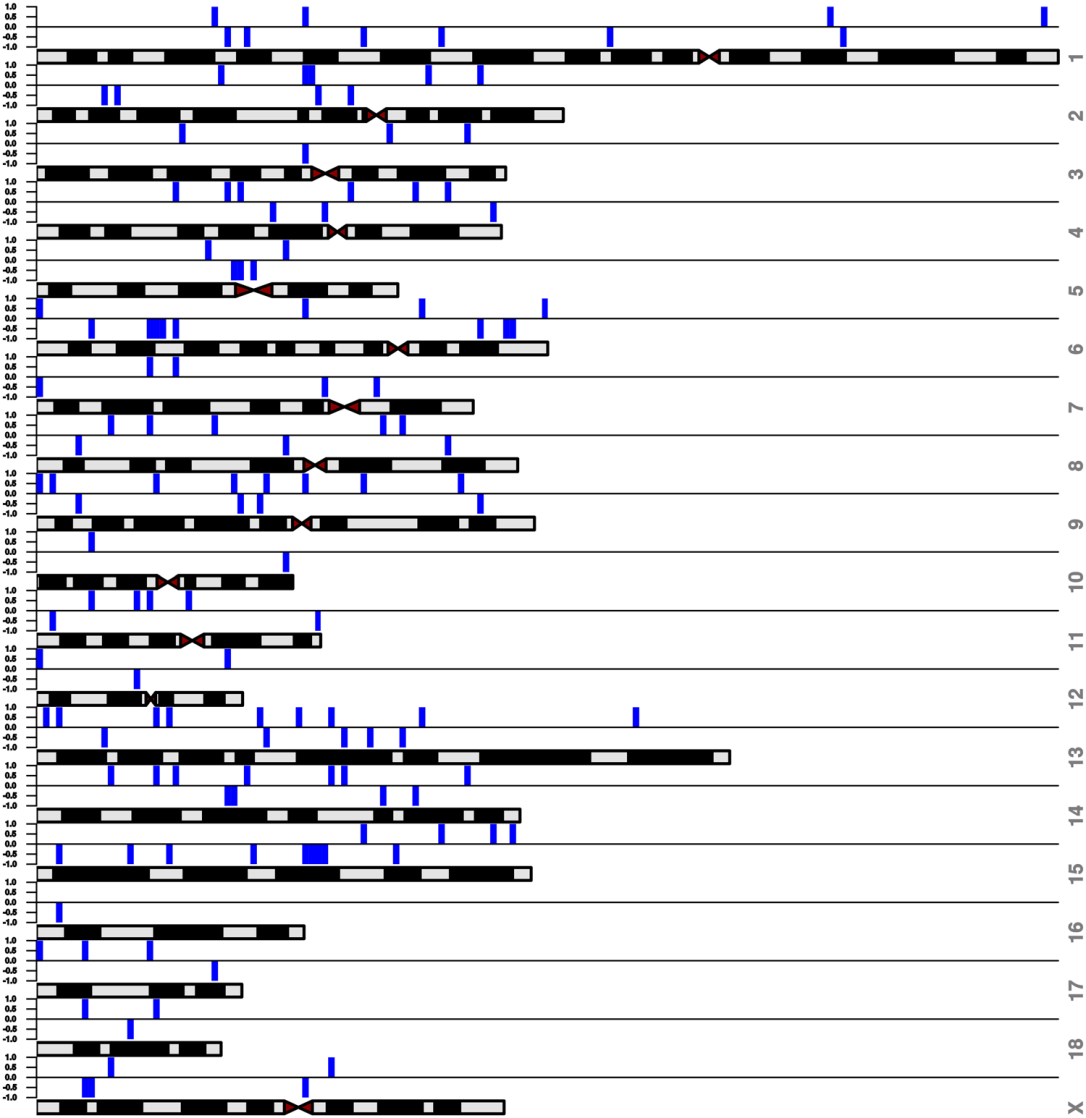
Duplication of mitochondrial genome in the nuclear genome. Blue bars represent the locations of duplicated mitochondrial genome against the porcine chromosomes.

Using BLASTN, the pig mitochondrial genome was compared to the pig nuclear genome. Many regions among 18 somatic chromosomes and the X chromosome of the porcine nuclear genome matched to the mitochondrial genome (Fig. 1).

### Comparison of mtDNA copy number

**Figure 2:**
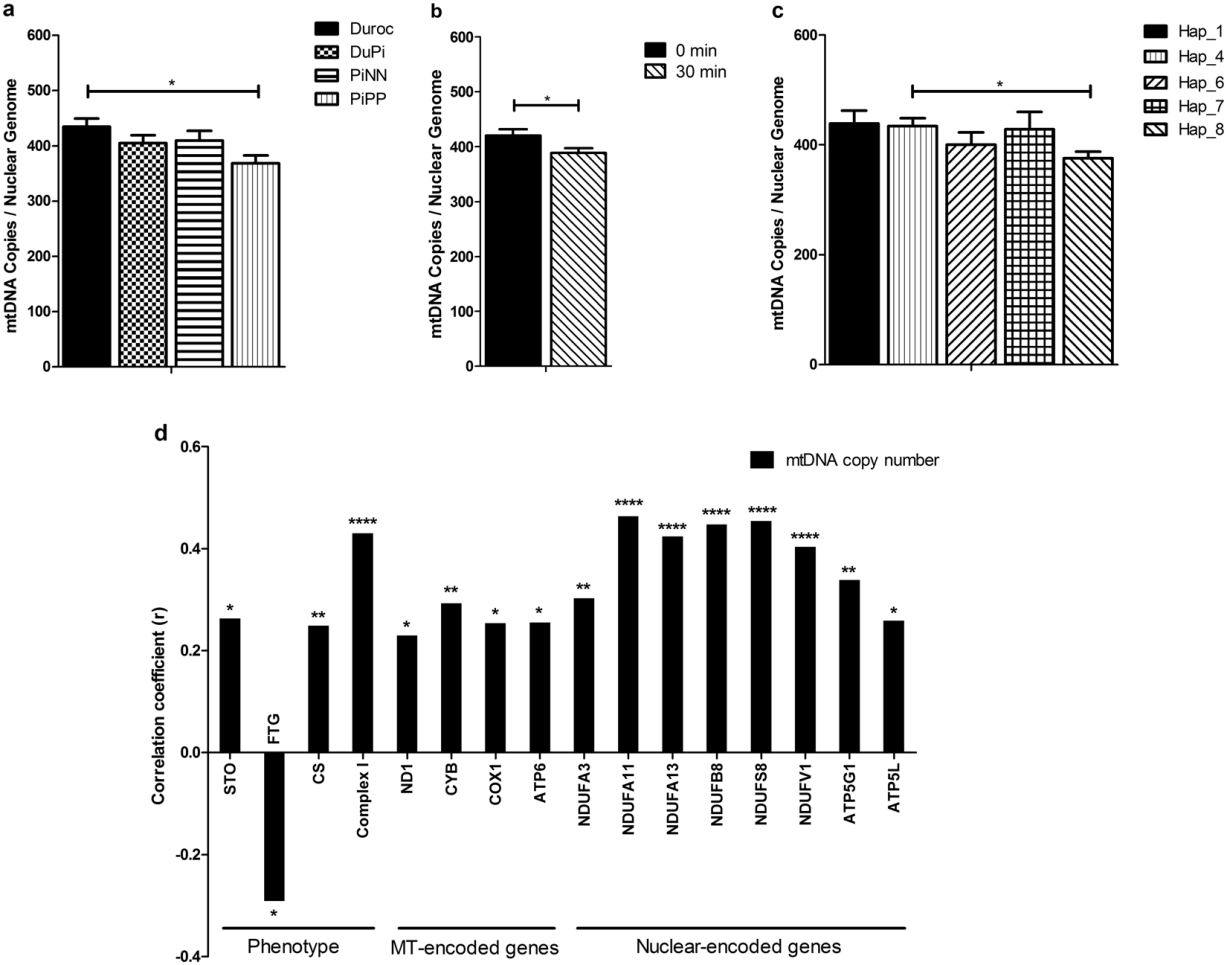
Absolute mitochondrial DNA copy number in porcine ***longissimus*** muscles. Least-square means with standard error (Lsmeans ± SE) of mtDNA copy number (**a**) in Duroc, DuPi, PiNN, and PiPP pigs (**b**) at 0 min and 30 min postmortem (**c**) in different mitochondrial haplotypes (**d**). The correlation coefficient of mtDNA copy number and OPXHOS gene expression with phenotypes of traits. *p < 0.05; **p < 0.01****p < 0.0001. See also Table S3 and S4.

PiPP pigs had the lowest mtDNA copy number (368 copies per nuclear genome) among the four breeds, especially compared to Duroc pigs (435 copies per nuclear genome, *p* = 0.02, Fig. 2a). Further, mtDNA copy number in longissimus muscle was decreased from 420 copies per nuclear genome at 0 min postmortem to 389 copies at 30 min postmortem (*p* = 0.01, Fig. 2b). Finally, muscle from pigs with haplotype 8 contained the lowest mtDNA copy number. Pigs with haplotype 8 had 375 copies per nuclear genome in their muscle tissue, which was significantly less than the 435 copies per nuclear genome seen in pigs with haplotype 4 (*p* = 0.02, Fig. 2c). There were no significant effects of sex on mtDNA copy number.

### Correlation between mtDNA copy number, phenotype, and gene expression

As shown in Fig. 2d, mtDNA copy number was significantly correlated with muscle fiber type (STO: r = 0.263 *p* = 0.031; FTG: r = −0.292 *p* = 0.017) and enzyme activities (CS: r = 0.249 *p* = 0.009; complex I: r = 0.43 *p* = < 0.0001). Moreover, the expression of mitochondria-encoded genes (*ND1*, *CYB*, *COX1*, and *ATP6*) and nuclear-encoded OXPHOS genes (*NDUFA3*, *NDUFA11*, *NDUFA13*, *NDUFB8*, *NDUFS8*, *NDUFV1*, *ATP5G1*, and *ATP5L*) were significantly correlated with mtDNA copy number (*p* values ranged from 0.006 to 0.031 and < 0.0001 to 0.015, respectively. MtDNA copy number was weakly correlated with PPARG coactivator 1 alpha (*PGC-1α)* mRNA (*p* = 0.156).

### Comparison of mitochondrial and nuclear encoded OXPHOS genes expression

To further examine breed and time differences in postmortem muscular energy metabolism at a molecular level, both mitochondrial and nuclear encoded gene expression was profiled at 0 and 30 min postmortem using qPCR in all four pig breeds. No mitochondrial-encoded genes were differently expressed between 0 and 30 min postmortem (Table 1). The mRNA levels of 4 nuclear-encoded genes, NDUFB8, COX7A2 and ATP5L were significantly lower at 30 min postmortem than 0 min (p values < 0.0001 to 0.02).

**Table 1:** OXPHOS gene expression at 0 and 30 min postmortem.

Gene		0 min (n = 58) Lsmean_SE_	30 min (n = 58) Lsmean_SE_	0 vs 30 min p-value
Mitochondrial-encoded	*ND1*	9.978_0.316_	10.126_0.401_	0.768
	*ND2*	7.528_0.416_	7.025_0.484_	0.433
	*ND4*	13.780_0.435_	13.449_0.669_	0.680
	*ND6*	6.641_0.316_	6.262_0.443_	0.462
	*CYB*	0.007_0.0004_	0.008_0.0005_	0.213
	*COX1*	20.677_0.580_	21.731_0.854_	0.306
	*ATP6*	17.539_0.658_	16.849_0.770_	0.507
Nuclear-encoded	*NDUFA3*	0.022_0.002_	0.024_0.002_	0.367
	*NDUFA11*	0.257_0.009_	0.264_0.008_	0.549
	*NDUFA13*	0.333_0.012_	0.336_0.010_	0.858
	*NDUFB8*	0.639_0.020_	0.348_0.013_	**<0.0001**
	*NDUFS8*	0.320_0.013_	0.304_0.011_	0.326
	*NDUFV1*	0.175_0.007_	0.188_0.006_	0.105
	*COX7A2*	0.132_0.006_	0.086_0.004_	**<0.0001**
	*ATP5G1*	0.521_0.025_	0.566_0.022_	0.071
	*ATP5L*	0.036_0.003_	0.029_0.002_	**0.016**

**Figure 3:**
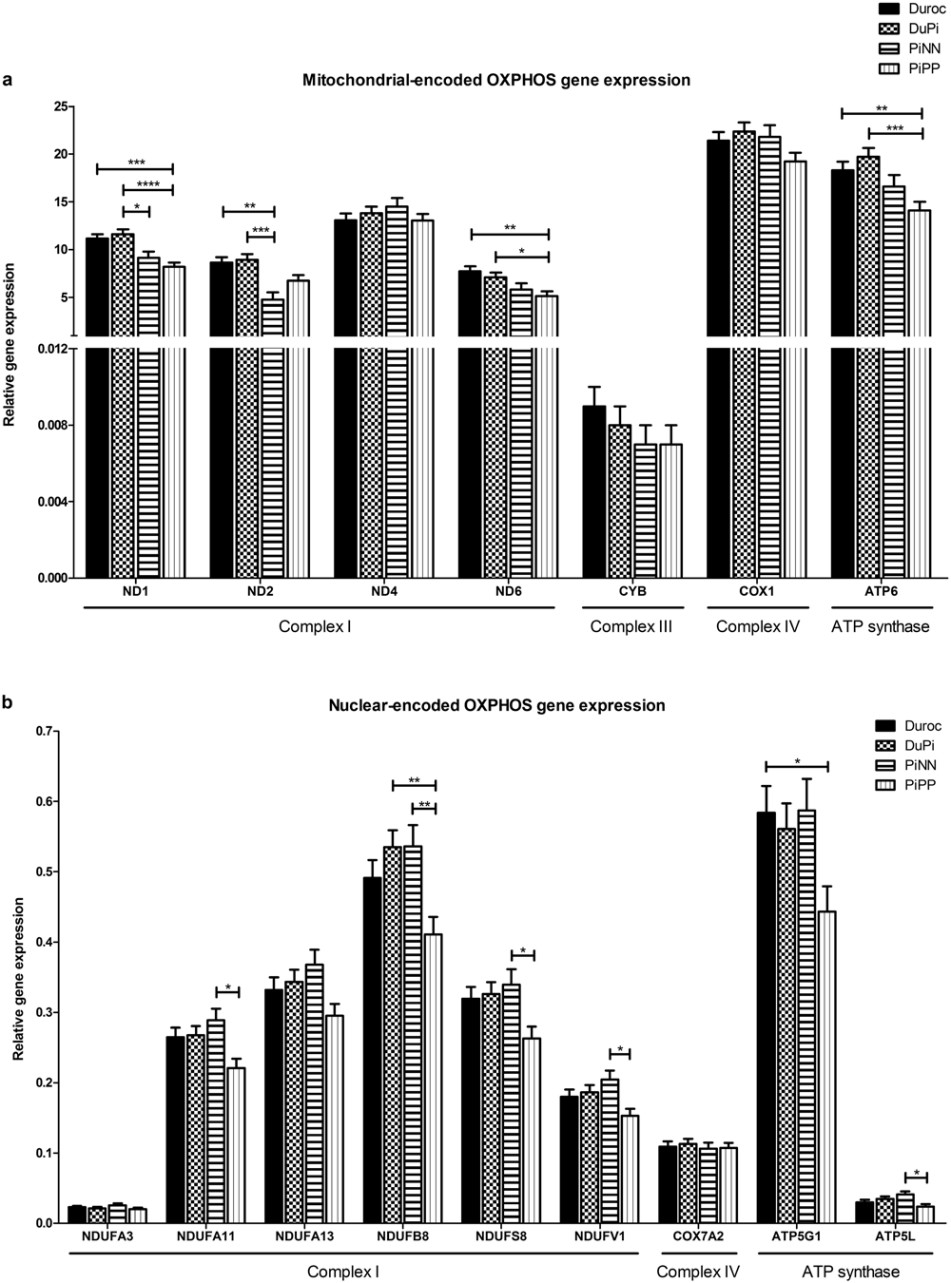
Relative mRNA expression of OXPHOS subunits in ***longissimus*** muscles of Duroc, DuPi, PiNN, and PiPP pigs. Least-square means with standard error (Lsmeans ± SE) of relative gene expressions for (**a**) mitochondrial-encoded complex I subunits (*ND1, ND2, ND4*, and *ND6*), complex III subunit (*CYB*), complex IV subunit (*COX1*) and ATP synthase subunit (*ATP6*) (**b**) nuclear-encoded complex I subunits (*NDUFA3, NDUFA11, NDUFA13, NDUFB8, NDUFS8*, and *NDUFV1*), complex IV subunit (*COX7A2*) and ATP synthase subunits (*ATP5G1* and *ATP5L*). Relative gene expression was normalized to reference genes *ACTB, RPL32* and *RPS11* using 2^(−∆Ct)^. *p < 0.05; **p < 0.01; ***p < 0.001; ****p < 0.0001. See also Table S3.

Out of the sixteen genes investigated, ten genes including mitochondrial-encoded *ND1, ND2, ND6, ATP6* were differently expressed between breeds (Fig. 3a). PiPP pigs had the most differentially expressed genes, especially compared to the Duroc and DuPi breeds: complex I subunits ND1 and ND6 and the ATP synthase subunit ATP6 were all significantly upregulated in Duroc pigs (p values < 0.0001 to 0.003). Nuclear-encoded *NDUFA11, NDUFB8, NDUFS8, NDUFV1, ATP5G1* and *ATP5L* showed significant differences among breeds (*p* < 0.05, Supplementary Table 2) (Fig. 3b). All six differentially expressed were down-regulated in PiPP pigs compared to the three other breeds.

Eleven genes including *ND1, ND2, ND6, CYB, COX1, ATP6* and nuclear-encoded *NDUFA11, NDUFA13, NDUFB8, ATP5G1* and *ATP5L* showed significant differences in haplotypes (Supplementary Table 3). Only three genes including nuclear-encoded *NDUFB8, COX7A2* and *ATP5L* demonstrated that they were significantly influenced by time. No gene expression was affected by sex.

**Figure 4:**
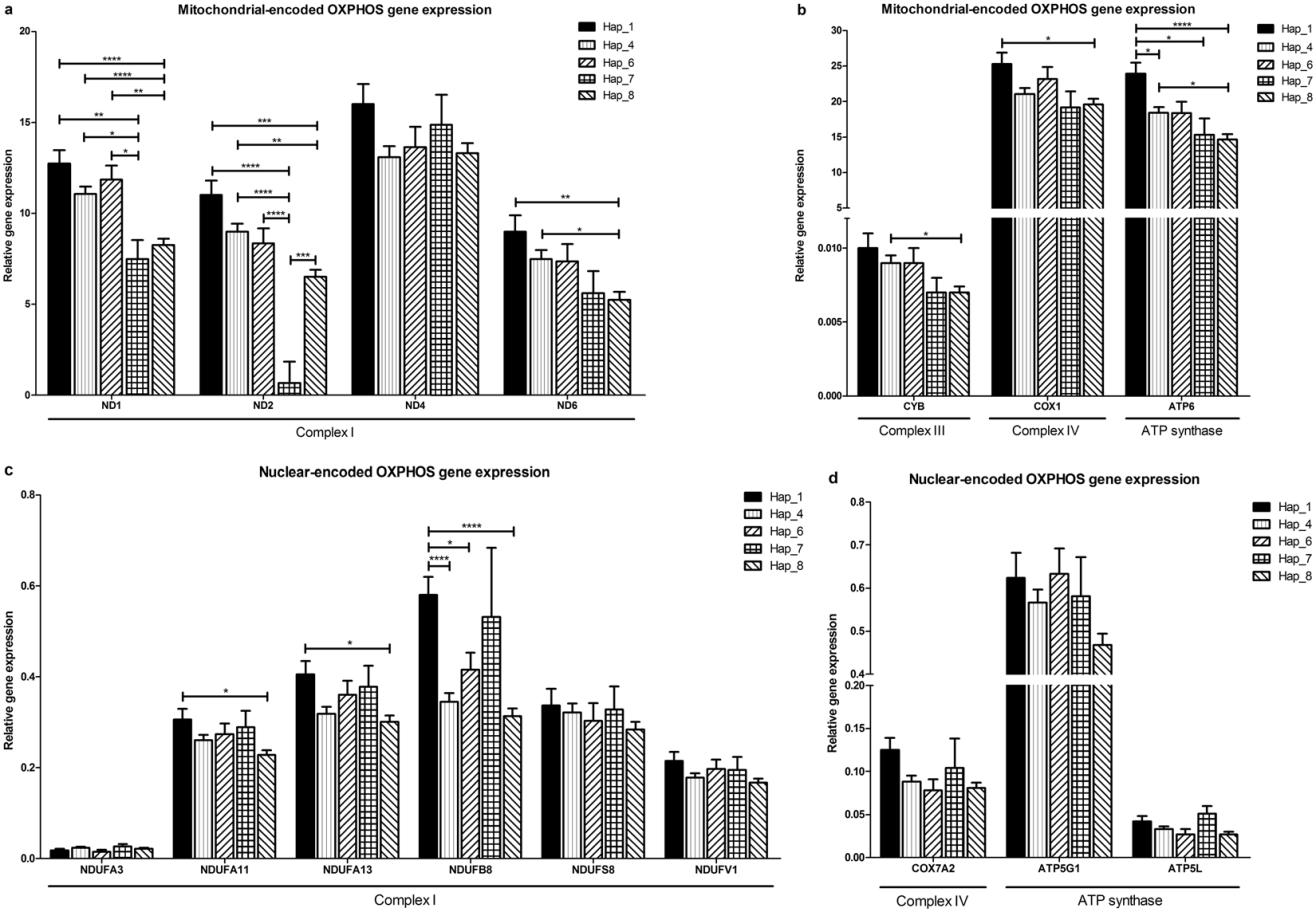
Relative mRNA expression of OXPHOS subunits in ***longissimus*** muscles of different mitochondrial haplotypes. Least-square means with standard error (Lsmeans ± SE) of relative gene expression for (**a**) mitochondrial-encoded complex I subunits (*ND1, ND2, ND4*, and *ND6*). (**b**) complex III subunit (*CYB*), complex IV subunit (*COX1*) and ATP synthase subunit (*ATP6*). (**c**) nuclear-encoded complex I subunits (*NDUFA3, NDUFA11, NDUFA13, NDUFB8, NDUFS8* and *NDUFV1*). (**d**) complex IV subunit (*COX7A2*) and ATP synthase subunits (*ATP5G1* and *ATP5L*). Relative gene expression was normalized to reference genes *ACTB, RPL32* and *RPS11* using 2^(−∆Ct)^. *p < 0.05; **p < 0.01; ***p < 0.001; ****p < 0.0001. See also Table S4.

All PiPP pigs had haplotype 8, which showed significantly lower gene expression than haplotypes 1, 4, and 6 for *ND1*, *ND2*, *ND6*, *CYB*, *COX1*, and *ATP6* (*p* values from *p* < 0.0001 to 0.048; Fig. 4a and b). Complex I subunits *NDUFA11*, *NDUFA13*, and *NDUFB8* showed significantly lower gene expression in haplotype 8 than haplotype 1 pigs (*p* values < 0.0001 to 0.029, Fig. 4c and d).

### Correlation between expression levels of mitochondrial, nuclear encoded genes and phenotype

**Figure 5:**
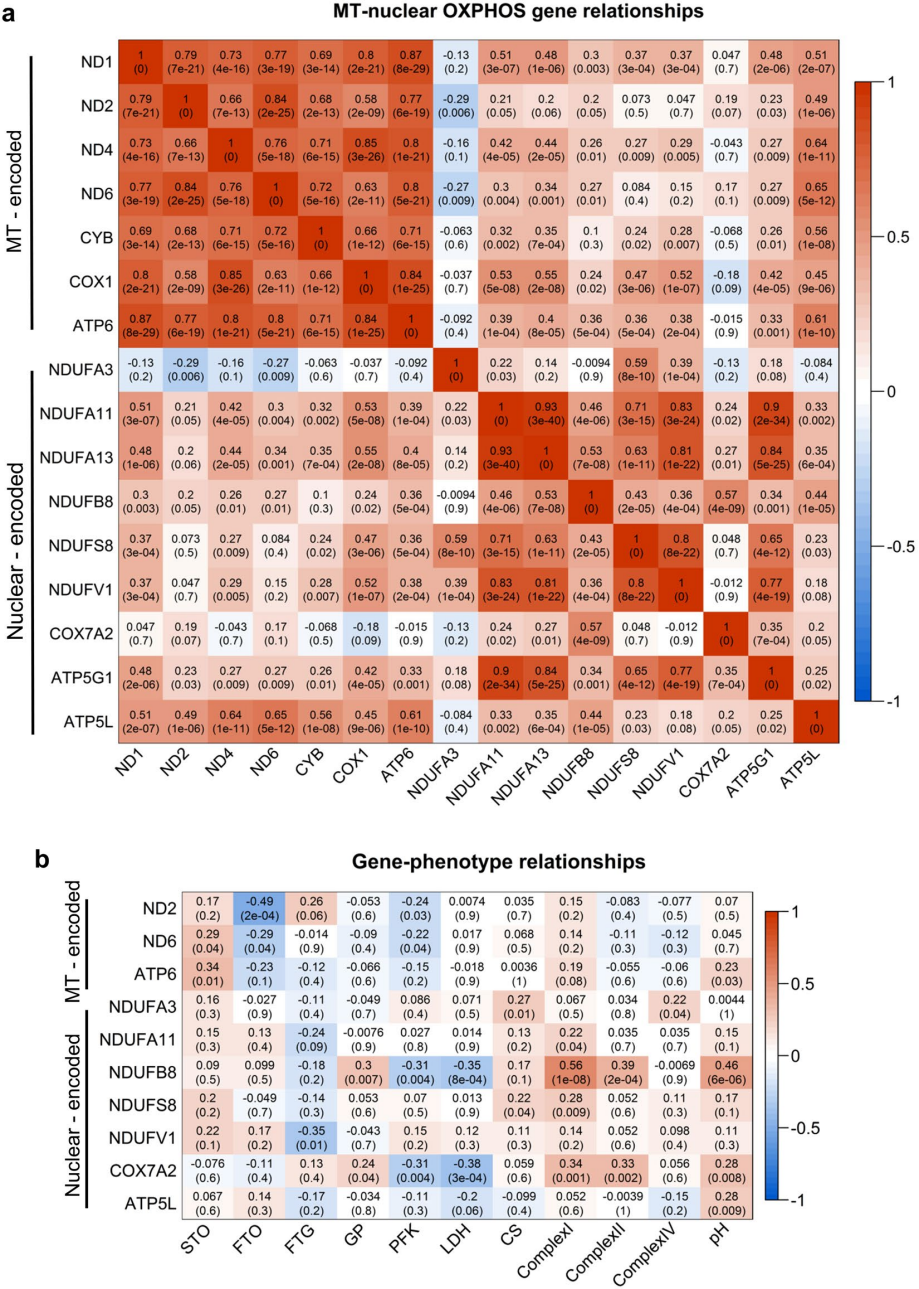
Correlation matrix of OXPHOS gene expression and phenotypes. (**a**) correlation matrix between mitochondrial and nuclear encoded OXPHOS gene expressions. (**b**) correlation matrix between OXPHOS gene expression and phenotype. Number in each cell represents the value of correlation coefficients and the corresponding p-values. Cell color indicates correlation (red, positive correlation; blue, negative correlation).

Pairwise correlation was calculated and plotted for each individual OXPHOS gene (Fig. 5a). All included genes were significantly correlated with at least one of the other genes. Further, all mitochondrial-encoded OXPHOS subunits tended to be more tightly co-expressed while nuclear-encoded OXPHOS subunits were in a tight co-expressed relationship. Figure 5b shows the correlation between the expression of OXPHOS genes and phenotype. The expression of mitochondrial encoded OXPHOS subunits *ND2*, *ND6*, and *ATP6* and the nuclear encoded subunit *NDUFV1* were highly correlated with muscle fiber type (*ND2*: FTO r = −0.49 *p* = 0.0002; *ND6*: STO r = 0.286 *p* = 0.042; *ATP6*: STO r = 0.342 *p* = 0.014; *NDUFV1*: FTG r = −0.346 *p* = 0.013). The nuclear encoded OXPHOS subunits *NDUFA3*, *NDUFA11*, *NDUFB8*, *NDUFS8*, and *COX7A2* were significantly correlated with at least one of the activities of oxidative enzymes CS, complex I, complex II, and complex IV. The expression of *ND2* (r = −0.239 *p* = 0.026) and *ND6* (r = −0.221 *p* = 0.04) were negatively correlated with PFK activity. The expression of *NDUFB8 and COX7A2* were highly correlated with the activities of GP (*NDUFB8*: r = 0.304 *p* = 0.007; COX7A2: r = 0.236 *p* = 0.037), PFK (*NDUFB8*: r = −0.309 *p* = 0.004; *COX7A2*: r = −0.305 *p* = 0.004), and LDH (*NDUFB8*: r = −0.354 *p* = 0.001; *COX7A2*: r = −0.376 p = 0.0003). The mRNA levels of *ATP6*, *NDUFB8*, *COX7A2*, and *ATP5L* were positively correlated with pH (*p* values < 0.0001 to 0.028).

The expression of the master regulator of mitochondrial biogenesis and oxidative phosphorylation *PGC-1a* was significantly correlated with the phenotypes of CS, complex I, complex II, complex IV, and pH (Table 2). The mRNA levels of *PGC-1a* were also significantly correlated with the expression of mitochondrial-encoded genes *ND1, ND2, ND4, ND6, CYB, COX1*, and *ATP6*. Among these genes, *ATP6, ND6*, and *ND2* showed the top three most significant correlations with *PGC-1a* (*ATP6*: r = 0.415 *p* < 0.0001; *ND6*: r = 0.372 *p* = 0.0003; *ND2*: r = 0.361*p* = 0.0004). The expression of *PGC-1a* showed a significant correlation with the nuclear-encoded genes *NUDFA11, NDUFA13, NDUFB8, COX7A2, ATP5G1*, and *ATP5L* (*p* values < 0.0001 to 0.036).

**Table 2:** Correlation between *PGC-1a*, phenotype, and OXPHOS gene expression.

Phenotype/mRNA		*PGC-1a*	
		r	p
Phenotype	CS	0.382	**0.0003**
	Complex I	0.405	**<0.0001**
	Complex II	0.234	**0.030**
	Complex IV	0.309	**0.004**
	pH	0.285	**0.007**
MT gene	*ND1*	0.346	**0.001**
	*ND2*	0.361	**0.0004**
	*ND4*	0.282	**0.007**
	*ND6*	0.372	**0.0003**
	*CYB*	0.246	**0.019**
	*COX1*	0.220	**0.036**
	*ATP6*	0.415	**<0.0001**
Nuclear gene	*NDUFA11*	0.266	**0.011**
	*NDUFA13*	0.220	**0.036**
	*NDUFB8*	0.341	**0.001**
	*COX7A2*	0.397	**<0.0001**
	*ATP5G1*	0.298	**0.004**
	*ATP5L*	0.358	**0.001**

## Discussion

We compared the mitochondrial DNA content, gene expression pattern of mitochondrial and nuclear encoded OXPHOS subunits, metabolic enzyme activities and mitochondrial respiration in four pig breeds distinct in muscle phenotype. The effect of mitochondrial haplotypes on mitochondrial DNA copy number and OXPHOS gene expression were also examined since there were different haplotypes among the investigated pig breeds.

Muscle samples were collected from the fat-type Duroc pigs with a higher proportion of STO fibers and greater oxidative enzyme activities. Pietrain pigs are muscular and lean, and their muscles contain more FTG fibers. DuPi pig is a Duroc-Pietrain F2 crossbred. Mutations within ryanodine receptor 11 (RYR1) are frequently detected in Pietrain pigs. This leads to abnormal Ca^2+^ homeostasis and result in increased excitability of the muscle associated with MHS. This genetic defect consequently leads to reduction of water holding capacity, loss of stress resistance and PSE meat^19–21^.

### Reduced mtDNA copy number in PiPP pigs

Previous studies have demonstrated the relative mtDNA copy number in porcine muscle^25,26^. In this study we quantified the absolute mtDNA copy number by qPCR and found that PiPP pigs contained the lowest amount of mtDNA copies in muscle cells compared to PiNN, DuPi, and particularly Duroc pigs. mtDNA copy number was positively correlated with STO fibers and negatively correlated with FTG fibers and indicated a strong association between mtDNA copy number and muscle fiber types.

The underlying cause of the mtDNA copy number variation between pig breeds remains unknown. In other case, changes in cytosolic Ca^2+^ and pH could influence the amount of ROS produced at the mitochondrial respiratory complex I and III^27^. The abnormal Ca^2+^ homeostasis in PiPP pigs may result in oxidative stress associated with elevated ROS production. The elevated ROS could increase mitophagy to remove damaged mitochondria and leads to mitochondrial degradation^28,29^. The copy number depletion associated with increased mitochondria turnover is caused by a burst of ROS production^30^. Indeed, the burst of ROS and mtDNA depletion have been observed in cell-culture experiments. All together, these may partially explain the copy number variation of mtDNA in pig breeds. However, supporting evidence under physiological situation is still needed.

### Decreased transcript levels of mitochondrial and nuclear encoded OXPHOS genes in PiPP pigs

Decreased abundance of many nuclear-encoded OXPHOS subunits transcripts was found in PiPP pigs compared to other breeds. This phenomenon is very likely caused by the abnormal Ca^2+^ homeostasis in PiPP pigs since MHS knock-in mice show a clear Ca^2+^ overload in the mitochondrial matrix and a switch to a compromised bioenergetics state characterized by low OXPHOS^31^. Similarly, transcript abundance of mitochondrial-encoded genes was decreased in PiPP pigs including subunits of complex I and ATP synthase. The previous study reported that ATP concentration has been significantly reduced in PiPP pigs compared to other breeds because of their accelerated energy consumption^14^. ATP sensing by transcriptional machinery has been proposed to regulate the initiation of mitochondrial transcription and promotor usage^32–34^. Therefore, the lower ATP availability in PiPP pigs may influence their mitochondrial transcription and function.

Recent work has established that glucocorticoid receptor (GR) is translocated from cytosol to the mitochondria and binds to the D-loop control region while stress and corticosteroids have a direct influence on hippocampal mtDNA gene expression in rats^35^. Since *RYR1* mutated PiPP pigs are stress susceptible, it is speculated that the abundance of mtDNA transcripts may be decreased in those pigs.

The early postmortem period had no effect on the expression of mitochondrial-encoded OXPHOS genes, but the mtDNA copy number was decreased from 0 min to 30 min postmortem. In other words, the early phase of hypoxia triggered mitochondrial genome instability while transcript abundance was maintained in porcine muscle. This disparity is of interest and might provide valuable information toward the understanding of mitochondrial mechanisms in muscle tissue under hypoxia, which has been linked to oxidative stress, ischemia, and cancer^36^. In addition, the identified postmortem mitochondrial properties are relevant to muscle injury, also caused by oxygen depletion^37^.

### The effect of haplotype on mtDNA copy number and OXPHOS gene expression

Our study showed for the first time the effect of different mitochondrial haplotypes on muscle fiber types and mitochondrial respiration at both the phenotypic and molecular level in pigs. Among different haplotypes, our results showed variations in complex I activity and mtDNA copy number. At the molecular level, six of the mitochondrial-encoded and three of the nuclear-encoded OXHPOS genes were differentially expressed. These findings suggest that mitochondrial haplotypes could contribute to variations in mitochondrial content and OXPHOS system function.

The mitochondrial haplotype has been found to affect mtDNA copy number, OXPHOS respiration, and mitochondrial molecular function in *Drosophila* ^1,7,38^. In porcine transmitochondrial cybrids, the mitochondrial haplotype is linked to metabolic traits including ROS production, ATP content, and complex II activity^39^. The assembly kinetics of OXPHOS complexes is proposed to be modulated by mitochondrial DNA background^40^.

### Coordination of the nuclear and mitochondrial genomes contribute to phenotype

Our results showed that all mitochondrial-encoded OXPHOS subunits were highly co-expressed; moreover also the nuclear-encoded OXPHOS subunits tended to be in a tight co-expressed relationship. This observation reflects the fact that the mitochondrial genome has its own transcription machinery distinct from the nuclear genome. Although mitochondrial- and nuclear-encoded OXPHOS genes are expressed separately via different transcription machineries in different cellular locations, they are not completely independent. The observed association between mitochondrial- and nuclear-encoded OXPHOS genes is in line with the theory of mitochondrial-nuclear crosstalk. PPARG coactivator 1 alpha (PGC-1α) is a nuclear-encoded transcription factor that acts as a master coordinator to mediate mitochondrial biogenesis and oxidative phosphorylation^41^. In this study, the mRNA level of *PGC-1α* significantly correlated with all investigated mitochondrial-encoded OXPHOS subunits, six nuclear-encoded subunits and enzyme activities of complex I, II, and IV. In fact, the nuclear-encoded subunits need to be imported into the mitochondria together with mitochondrial-encoded subunits to form a fully assembled functional OXPHOS system in the mitochondrial inner membrane. Accordingly, the absence of the mtDNA-encoded subunits COX1 and COX2 has been shown to affect the stability of some subunits of nuclear encoded respiratory chain proteins^42^.

In our results, different mitochondrial haplotypes showed variation of the expression of mitochondrial-encoded subunits *ND2*, *ND6* and *ATP6* as well as nuclear-encoded subunits *NDUFA11* and *NDUFB8*. These genes were co-expressed and not only highly correlated to *PGC-1α* but also highly associated with different muscle fiber types and enzyme activities of complex I and II. Therefore, we propose that mitochondrial haplotype contributes to muscle fiber type and energy metabolism in porcine via altering the gene expression of OXPHOS subunits mediated by the nuclear-mitochondrial crosstalk. In addition, different haplotypes showed variation in complex I activity. It directly supported the link between haplotype and energy metabolism.

The disrupted Ca^2+^ homeostasis affects mitochondrial membrane biogenesis and hence metabolic stress^43^. Under conditions of oxidative stress, the expression of *ND6* is suppressed through methylation^44^. Consistent with our results, downregulated *ND6* expression in PiPP pigs has been associated with mutant *RYR1*-induced mitochondrial injury and oxidative stress^45^. These evidences supported the possibility of haplotype 8 could be linked to oxidative stress with altered mitochondrial transcription in PiPP pigs.

Our results showed some of the mitochondrial and nuclear-encoded OXPHOS transcripts were significantly correlated to at least one of the phenotypes including muscle fiber type, metabolic enzyme activities and pH. The mtDNA encoded OXPHOS gene expression was highly associated with muscle fiber types, which is consistent with the fact that STO muscle fibers in general contain more mitochondria^46^. It is worth mentioning that the mRNA levels of *ATP6, ND6*, and *ND2*, which were the top three genes correlated with *PGC-1α*, also significantly correlated with muscle fiber type. Hence, it raised the possibility that mitochondrial-encoded subunits with high correlation with *PGC-1a* showed effects on muscle fiber types. The muscle oxidative capacity not only relies on mitochondrial function but also on mitochondrial density^46^. Our measured mtDNA copy number was correlated positively with STO fibers and oxidative enzyme activity while negatively correlated with FTG fibers. The mtDNA copy number is related to mitochondrial oxidative capacity and adipocyte lipogenesis^47^.

## Conclusions

In summary, we investigated the mitochondrial DNA content and the expression of both mitochondrial and nuclear encoded OXPHOS genes in conjunction with post-mortem muscle phenotype, and metabolic enzyme activities in distinct haplotypes of pig breeds including Duroc, DuPi, PiNN, and PiPP. The most significant link between haplotypes or breeds to the muscle phenotype was found between the muscle fibers type and complex I. Specific expression pattern of mt transcript including ND1, ND6, and ATP6 and nuclear-encoded subunits NDUFA11 and NDUFB8 was identified and in turn play a role in muscle fibers type and enzyme activities of complex I. All of these changes in the PiPP haplotype 8 pigs may partially contribute to negative outcomes of meat quality such as pale soft exudative pork. PiPP pigs showed the lowest mtDNA copy number and reduced gene expression of many mitochondrial and nuclear encoded OXPHOS subunits compared to the other breeds. Our results provide valuable information on haplotype and breed-specific mitochondrial content variation as well as the molecular basis of mitochondrial respiration. Haplotypes could be linked to porcine energy metabolism at a functional level by altering gene expression of mitochondrial and nuclear OXPHOS subunits. Since haplotype 7 Pietrain pigs demonstrate a high ratio of FTO muscle fibers, while haplotype 8 pigs show the lowest complex I activity among all other haplotypes, implementing a selection of the favorable haplotype 7 along with the RYR1 locus in marker assisted selection program may further improve meat quality. Selection of the favorable haplotype can be used in marker assisted selection in pig breeding strategy.

## Material and Methods

### Sample collection and phenotypic measurement

The experiment and muscle collection were approved and authorized by the German and European animal welfare regulations for animal husbandry, transport, and slaughter^12–14^. All experimental procedures, including animal care and tissue sample collection, followed guidelines for safeguarding and good scientific practice in accordance with the German Law of Animal Protection, officially authorized by the Animal Care Committee and authorities [Niedersächsischen Landesamt für Verbraucherschutz und Lebensmittelsicherheit (LAVES) 33.42502/01-47.05].

Duroc, PiNN, PiPP, and DuPi pigs were raised to the age of 180 days at the University of Bonn. Muscle samples from each breed (Duroc, n = 15; DuPi, n = 16; PiNN, n = 12; PiPP, n = 15) were collected immediately (0 min postmortem) and 30 min after stunning (30 min postmortem) from LM between the 13th and 14th thoracic vertebrae. Samples were frozen in liquid nitrogen and stored at −80 °C until analysis.

We used the samples and all phenotypical traits from previously study which were measured as described^12–14^. In brief, the cryopreserved muscle samples were cutting into slices of 12 µm thickness. NADH tetrazolium reductase and Myofibrillar ATPase were stained to identify the muscle fiber types. 3 sections were used for calculating the percentage of the slow-twitch-oxidative (STO), fast-twitch-oxidative (FTO) and fast-twitch glycolytic (FTG) fibers by relating the number of counted fibers of each type to the total counted fiber number. For measurement of Metabolic Enzyme Activities, muscle samples were homogenized and all the experiments were finished within 2 h in duplicate. GP catalyzed the degradation of glycogen (2 mg/ml) to glucose-1-phosphate followed by the isomerization to glucose-6-phosphate (G-6-P) 2. GP activity was determined spectrophotometrically by the reduction of NADP+ (1.6 mM) to NADPH at 340 nm and pH 6.8 when G-6-P dehydrogenase (5 U/ml) catalyzed G-6-P to gluconate-6-phosphate. PFK catalyzes fructose-6-phosphate (3.0 mM) to fructose 1,6-bis-phosphate, which is split to glyceraldehyde-3-phosphate and dihydroxyacetonephosphate (DHAP). PFK activity was determined by the oxidation of NADH (1.6 mM) to NAD + at 340 nm and pH 8.0 when glycerol-3-phosphate dehydrogenase (10 U/ml) /triosephosphate isomerase (100 U/ml) catalyzed DHAP to glycerol-3-phosphate. LDH activity was determined by the oxidation of NADH (150 µM) to NAD+ at 340 nm when LDH catalyzed pyruvate (1.2 mM) to lactate. CS catalyzes acetyl-CoA (0.1 mM) and oxaloacetate (0.5 mM) to citrate to liberate CoA. CS activity was determined by the irreversible reaction of CoA with 5,5′-Dithiobis-(2-nitrobenzoic acid; 0.1 mM) to thionitrobenzoic acid at 412 nm 3. Complex I was spectrophotometrically determined by following the oxidation of NADH (0.2 mM) to NAD+ at 340 nm. Complex II was determined at 600 nm following the reduction of 2, 6-dichlorophenolindophenol (DCPIP) by ubiquinol resulting from this reaction. Complex IV was determined by following the oxidation of reduced cytochrome c to the oxidized form at 550 nm and pH7.0.

### DNA and RNA extraction

Genomic DNA from LM samples was extracted. Total RNA was isolated from muscle samples using Tri-reagent and RNeasy Minikit (Qiagen, Hilden, Germany) with an on-column DNase treatment according to the manufacturer’s protocol. RNA integrity was assessed by 1% agarose gel electrophoresis. DNA and RNA concentration was measured on a NanoDrop ND-1000 spectrophotometer.

### Mitochondria-specific primer design

Primers for the detection of mitochondrial DNA (mtDNA) copy number were carefully designed avoiding co-amplification of mitochondrial duplicated regions in the nuclear genome. Duplication of the mitochondrial genome in the nuclear genome was detected using BLASTN (http://www.ncbi.nlm.nih.gov)^48^. The mitochondrial sequence (Sus scrofa 10.2 download from NCBI: http://www.ncbi.nlm.nih.gov/ on 1.9.2015) was split into fragments of 150 bps in length with a 50 bps overlap and blasted against the reference genome to identify a ‘unique’ mitochondrial sequence based on a significant threshold of E-value < 0.1 and length >100 bps. The result of duplicated regions was demonstrated using R package IdeoViz^49^ and a cytogenetic map of pig chromosomes was extracted from ArkDB (http://www.thearkdb.org/arkdb)^50^.

### Absolute quantification of mtDNA copy number

The absolute quantification approach was used to determine mtDNA copy number. The mitochondrial genes ND1, ND2, and COX1 were used to quantify mtDNA copy number, whereas the nuclear gene glucagon gene (GCG), which is highly conserved between species and presents as a single copy, was used as the single-copy reference gene^25,51^. Primer sequences were presented in Supplementary Table 4 online. Mitochondrial and nuclear DNA standards were prepared separately using PCR products in seven serial dilutions at a dilution factor of 10. The amplified DNA fragments were purified with the QIAquick PCR Purification kit (Qiagen, Hilden, Germany). The purified products were quantified using a NanoDrop ND-1000 spectrophotometer (Peqlab, Erlangen, Germany). The copy number was calculated according to the following equation^52^:

1 $$copies/\mu l=\frac{{\rm{ng}}/\mu l}{{\rm{m}}}$$

2 $$m=n\times [1.096\times {10}^{-12}]$$

where m is the mass of a single copy and n is the target size in base pairs.

The absolute copy numbers of *ND1*, *ND2*, *COX1* and *GCG* were calculated based on their standard curves using the following equation:

3 $$copies={10}^{(Ct-b)/a}$$

where a is the slope and b is the intercept of the regression line.

Since *GCG* is a single copy nuclear gene, the mtDNA copies per nuclear genome were calculated as follows:

4 $$mtDNA\,copies/nuclear\,genome=\frac{mtDNA\,copies}{nuclearDNA\,copies\,}$$

The mtDNA copy number per nuclear genome was calculated separately using *ND1*, *ND2*, and *COX1*. The data was reported as a mean.

### Measurement of gene expression

High-throughput gene-expression analysis with EvaGreen dye on a BioMark HD real-time PCR system was used to measure gene expression according to manufacturer’s recommendations (Fluidigm, San Francisco, CA, USA). All reagents were purchased from Fluidigm unless otherwise indicated. Briefly, cDNA was synthesized from 2 µg of total RNA using Superscript II reverse transcriptase and oligo-dT (Invitrogen) with specific target amplification and exonuclease I (New England Biolabs) treatment. qPCR reactions were performed using a 96 × 96 dynamic array and integrated fluidic circuit. Each sample inlet was loaded with 2.5 µL of 2 × SsoFast EvaGreen supermix with low ROX (Biorad), 0.25 µL of 20×DNA-binding dye sample loading reagent, and 2.25 µL of specific target amplification and exonuclease-I-treated sample. Assays were performed for mitochondrial-coded complex I subunits *ND1, ND2, ND3*, and *ND4*, complex III subunit *CYB*, complex IV subunit *COX1*, ATP synthase subunit *ATP6*, and nuclear-encoded complex I subunits *NDUFA3, NDUFA11, NDUFA13, NDUFB8, NDUFS8*, and *NDUFV1*, complex IV subunit *COX7A2*, ATP synthase subunits *ATP5G1* and *ATP5L*, and master regulator *PGC-1α*. All measurements were performed in duplicate. Primer sequence information is available in Supplementary Table 4. Reference genes *ACTB, RPL32*, and *RPS11* were used to normalize expression values.

### Sequence analysis

DNA from muscle samples of 53 animals (Duroc: N = 15, DuPi: N = 15, PiNN: N = 9, PiPP: N = 14) were sequenced using an ABI 3500 sequencer (Applied Biosystems Inc, Foster City, CA, USA). The D loop region was amplified using forward primer 5′-CTCCGCCATCAGCACCCAAAG-3′ and reverse primer 5′-GCACCTTGTTTGGATTRTCG-3′ ^53^. All sequences were aligned using Clustal × 2.1^54^. DNASP 5.1 software was used to analyze the haplotypes of all sequences^55^. The detailed information of haplotypes is shown in Supplementary Table 2. Only the haplotypes with at least three animals were included in the subsequent statistical analysis.

### Statistical analysis

Data were analyzed using SAS 9.4 statistical software (SAS Institute) and the MIXED procedure. The statistical model included the effects of breed (Duroc, PiNN, PiPP, and DuPi), sex (male and female), time (0 and 30 min postmortem). With the same model, we also calculated with haplotype (Haplotypes 1, 4, 6, 7, and 8) instead of breeds. The model was combined with a repeated statement for the time component to take into account correlations among measurements made on the same animal at time 0 and 30 min postmortem. Post hoc Tukey–Kramer method was used for multiple comparison adjustments. Results were reported as least-squares means (Lsmeans) with standard error (SE) and considered to be statistically significant if *p* < 0.05. Data were plotted using GraphPad Prism 5. The correlation coefficient (r) between gene expression and phenotypic measurement was calculated for all individuals. The correlation plots were generated in R.

## Electronic supplementary material

Supplementary Figure 1-4

Supplementary Table 1-4

## References

[1] Camus MF, Wolf JB, Morrow EH, Dowling DK (2015). Single Nucleotides in the mtDNA Sequence Modify Mitochondrial Molecular Function and Are Associated with Sex-Specific Effects on Fertility and Aging. Curr Biol.

[2] Shi Y, Buffenstein R, Pulliam DA, Van Remmen H (2010). Comparative studies of oxidative stress and mitochondrial function in aging. Integr Comp Biol.

[3] Baykara O, Sahin SK, Akbas F, Guven M, Onaran I (2016). The effects of mitochondrial DNA deletion and copy number variations on different exercise intensities in highly trained swimmers. Cell Mol Biol (Noisy-le-grand).

[4] Falah M (2016). The potential role for use of mitochondrial DNA copy number as predictive biomarker in presbycusis. Ther Clin Risk Manag.

[5] Gao Y (2016). Changes of the mitochondrial DNA copy number and the antioxidant system in the PBMC of hepatocellular carcinoma. Chinese journal of applied physiology.

[6] Huang J (2016). Decreased Peripheral Mitochondrial DNA Copy Number is Associated with the Risk of Heart Failure and Long-term Outcomes. Medicine (Baltimore).

[7] Salminen, T. S. *et al*. Mitochondrial genotype modulates mtDNA copy number and organismal phenotype in Drosophila. *Mitochondrion* (2017). 10.1016/j.mito.2017.02.001 28214560

[8] Tsai T, St John JC (2016). The role of mitochondrial DNA copy number, variants, and haplotypes in farm animal developmental outcome. Domest Anim Endocrinol.

[9] Picard M, Hepple RT, Burelle Y (2012). Mitochondrial functional specialization in glycolytic and oxidative muscle fibers: tailoring the organelle for optimal function. Am J Physiol Cell Physiol.

[10] Greaser ML, Cassens RG, Briskey EJ, Hoekstra WG (1969). Post-Mortem Changes in Subcellular Fractions from Normal and Pale, Soft, Exudative Porcine Muscle. 1. Calcium Accumulation and Adenosine Triphosphatase Activities. Journal of Food Science.

[11] Scheffler TL, Matarneh SK, England EM, Gerrard DE (2015). Mitochondria influence postmortem metabolism and pH in an *in vitro* model. Meat science.

[12] Werner C, Natter R, Schellander K, Wicke M (2010). Mitochondrial respiratory activity in porcine longissimus muscle fibers of different pig genetics in relation to their meat quality. Meat science.

[13] Werner C, Natter R, Wicke M (2010). Changes of the activities of glycolytic and oxidative enzymes before and after slaughter in the longissimus muscle of Pietrain and Duroc pigs and a Duroc-Pietrain crossbreed. Journal of animal science.

[14] Krischek C, Natter R, Wigger R, Wicke M (2011). Adenine nucleotide concentrations and glycolytic enzyme activities in longissimus muscle samples of different pig genotypes collected before and after slaughter. Meat science.

[15] Wimmers K (2008). Relationship between myosin heavy chain isoform expression and muscling in several diverse pig breeds. Journal of animal science.

[16] Essen-Gustavsson B, Karlsson A, Lundstrom K, Enfalt AC (1994). Intramuscular fat and muscle fibre lipid contents in halothane-gene-free pigs fed high or low protein diets and its relation to meat quality. Meat science.

[17] Karlsson AH, Klont RE, Fernandez X (1999). Skeletal muscle fibres as factors for pork quality. Livestock Production Science.

[18] Karlsson A, Essen-Gustavsson B, Lundstrom K (1994). Muscle glycogen depletion pattern in halothane-gene-free pigs at slaughter and its relation to meat quality. Meat science.

[19] Yue G (2003). Linkage and QTL mapping for Sus scrofa chromosome 6. Journal of Animal Breeding and Genetics.

[20] Shen QW, Underwood KR, Means WJ, McCormick RJ, Du M (2007). The halothane gene, energy metabolism, adenosine monophosphate-activated protein kinase, and glycolysis in postmortem pig longissimus dorsi muscle. Journal of animal science.

[21] Fujii J (1991). Identification of a mutation in porcine ryanodine receptor associated with malignant hyperthermia. Science.

[22] Liu X (2015). Muscle Transcriptional Profile Based on Muscle Fiber, Mitochondrial Respiratory Activity, and Metabolic Enzymes. Int J Biol Sci.

[23] Liu X (2016). MicroRNA-mRNA regulatory networking fine-tunes the porcine muscle fiber type, muscular mitochondrial respiratory and metabolic enzyme activities. BMC Genomics.

[24] Liu X (2016). Molecular changes in mitochondrial respiratory activity and metabolic enzyme activity in muscle of four pig breeds with distinct metabolic types. J Bioenerg Biomembr.

[25] Xie YM (2015). Quantitative changes in mitochondrial DNA copy number in various tissues of pigs during growth. Genet Mol Res.

[26] Shen L (2015). Transcriptome Analysis of Liangshan Pig Muscle Development at the Growth Curve Inflection Point and Asymptotic Stages Using Digital Gene Expression Profiling. PLoS One.

[27] Lindsay DP, Camara AK, Stowe DF, Lubbe R, Aldakkak M (2015). Differential effects of buffer pH on Ca(2+) -induced ROS emission with inhibited mitochondrial complexes I and III. Front Physiol.

[28] Lana A, Zolla L (2015). Apoptosis or autophagy, that is the question: Two ways for muscle sacrifice towards meat. Trends in Food Science & Technology.

[29] Dagda RK, Zhu J, Kulich SM, Chu CT (2008). Mitochondrially localized ERK2 regulates mitophagy and autophagic cell stress: implications for Parkinson’s disease. Autophagy.

[30] Fukuoh A (2014). Screen for mitochondrial DNA copy number maintenance genes reveals essential role for ATP synthase. Mol Syst Biol.

[31] Giulivi C (2011). Basal bioenergetic abnormalities in skeletal muscle from ryanodine receptor malignant hyperthermia-susceptible R163C knock-in mice. J Biol Chem.

[32] Bonawitz ND, Clayton DA, Shadel GS (2006). Initiation and beyond: multiple functions of the human mitochondrial transcription machinery. Mol Cell.

[33] Zollo O, Tiranti V, Sondheimer N (2012). Transcriptional requirements of the distal heavy-strand promoter of mtDNA. Proceedings of the National Academy of Sciences of the United States of America.

[34] Amiott EA, Jaehning JA (2006). Mitochondrial transcription is regulated via an ATP “sensing” mechanism that couples RNA abundance to respiration. Mol Cell.

[35] Hunter RG (2016). Stress and corticosteroids regulate rat hippocampal mitochondrial DNA gene expression via the glucocorticoid receptor. Proceedings of the National Academy of Sciences of the United States of America.

[36] Semenza GL (2001). Hypoxia-inducible factor 1: oxygen homeostasis and disease pathophysiology. Trends Mol Med.

[37] Ponsuksili S (2013). Correlated mRNAs and miRNAs from co-expression and regulatory networks affect porcine muscle and finally meat properties. BMC Genomics.

[38] Wolff JN (2016). Evolutionary implications of mitochondrial genetic variation: mitochondrial genetic effects on OXPHOS respiration and mitochondrial quantity change with age and sex in fruit flies. J Evol Biol.

[39] Yu G (2015). Mitochondrial Haplotypes Influence Metabolic Traits in Porcine Transmitochondrial Cybrids. Sci Rep.

[40] Pello R (2008). Mitochondrial DNA background modulates the assembly kinetics of OXPHOS complexes in a cellular model of mitochondrial disease. Hum Mol Genet.

[41] LeBleu VS (2014). PGC-1alpha mediates mitochondrial biogenesis and oxidative phosphorylation in cancer cells to promote metastasis. Nat Cell Biol.

[42] Marusich MF (1997). Expression of mtDNA and nDNA encoded respiratory chain proteins in chemically and genetically-derived Rho0 human fibroblasts: a comparison of subunit proteins in normal fibroblasts treated with ethidium bromide and fibroblasts from a patient with mtDNA depletion syndrome. Biochim Biophys Acta.

[43] Biswas G (1999). Retrograde Ca2+ signaling in C2C12 skeletal myocytes in response to mitochondrial genetic and metabolic stress: a novel mode of inter-organelle crosstalk. EMBO J.

[44] Shock LS, Thakkar PV, Peterson EJ, Moran RG, Taylor SM (2011). DNA methyltransferase 1, cytosine methylation, and cytosine hydroxymethylation in mammalian mitochondria. Proceedings of the National Academy of Sciences of the United States of America.

[45] Jin O (2014). RyR1 mutation associated with malignant hyperthermia facilitates catecholaminergic stress-included arrhythmia via mitochondrial injury and oxidative stress (893.8). The FASEB Journal.

[46] Gueguen N, Lefaucheur L, Fillaut M, Vincent A, Herpin P (2005). Control of skeletal muscle mitochondria respiration by adenine nucleotides: differential effect of ADP and ATP according to muscle contractile type in pigs. Comp Biochem Physiol B Biochem Mol Biol.

[47] Kaaman M (2007). Strong association between mitochondrial DNA copy number and lipogenesis in human white adipose tissue. Diabetologia.

[48] Altschul SF, Gish W, Miller W, Myers EW, Lipman DJ (1990). Basic local alignment search tool. J Mol Biol.

[49] IdeoViz: Plots data (continuous/discrete) along chromosomal ideogram v. R package version 1.6.0 (2014).

[50] Hu J (2001). The ARKdb: genome databases for farmed and other animals. Nucleic Acids Res.

[51] Wang J (2012). A genome-wide detection of copy number variations using SNP genotyping arrays in swine. BMC Genomics.

[52] Chan SW, Chevalier S, Aprikian A, Chen JZ (2013). Simultaneous quantification of mitochondrial DNA damage and copy number in circulating blood: a sensitive approach to systemic oxidative stress. Biomed Res Int.

[53] Jin L (2012). Mitochondrial DNA evidence indicates the local origin of domestic pigs in the upstream region of the Yangtze River. PLoS One.

[54] Thompson JD, Gibson TJ, Plewniak F, Jeanmougin F, Higgins DG (1997). The CLUSTAL_X windows interface: flexible strategies for multiple sequence alignment aided by quality analysis tools. Nucleic Acids Res.

[55] Librado P, Rozas J (2009). DnaSPv5: a software for comprehensive analysis of DNA polymorphism data. Bioinformatics.

