# Supplementary material for: Mitochondrial-nuclear crosstalk, haplotype and copy number variation distinct in muscle fiber type, mitochondrial respiratory and metabolic enzyme activities
Source: Sci Rep. 2017 Oct 25;7:14024. doi: 10.1038/s41598-017-14491-w (PMC5656670; doi:10.1038/s41598-017-14491-w)
Supplement: Supplementary file 1 — Supplementary Figure 1-4 [file 41598_2017_14491_MOESM1_ESM.pdf]

Mitochondrial-nuclear crosstalk, haplotype and copy number variation distinct in muscle fiber type, mitochondrial respiratory and metabolic enzyme activities

Xuan Liu[1], Nares Trakooljul[2], Frieder Hadlich[1], Eduard Murani[2], Klaus Wimmers[2] and Siriluck Ponsuksili [1],*

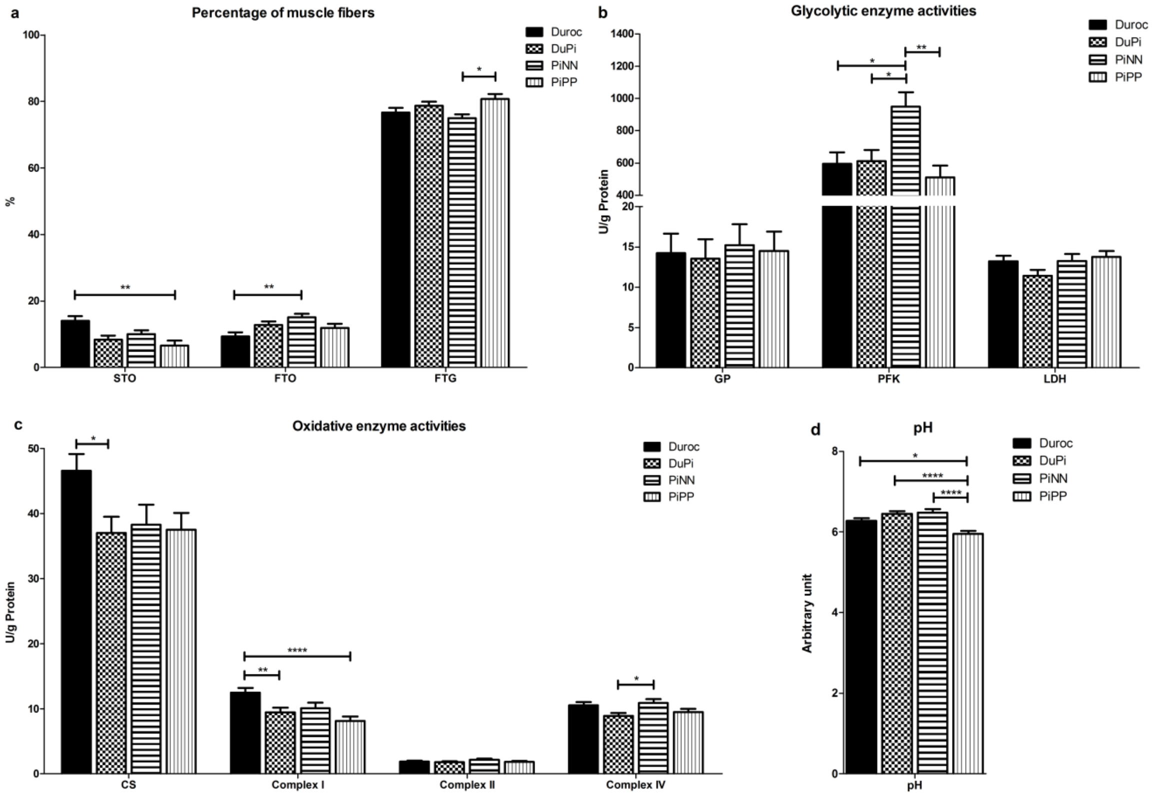

Supplementary Figure 1.

Phenotypes in longissimus muscles of Duroc, DuPi, PiNN and PiPP pig breeds. Least-square means with standard error (Lsmeans ± SE) of (A) percentage of muscle fibers. (B) Glycolytic enzyme activities. (C) Oxidative enzyme activities (D) pH. *p<0.05; **p<0.01; ****p<0.0001.

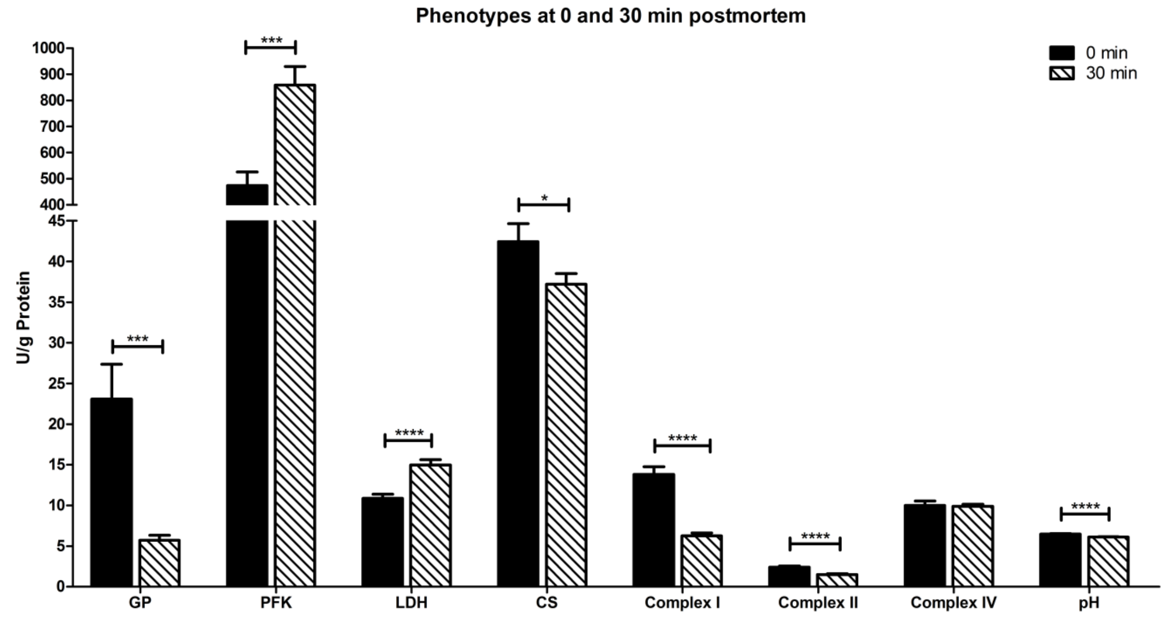

Supplementary Figure 2.

Phenotypes in longissimus muscles at 0 and 30 min postmortem. Least-square means with standard error (Lsmeans ± SE) of the enzyme activities of glycogen phosphorylase (GP), phosphofructokinase (PFK), lactate dehydrogenase (LDH), citrate synthase (CS), complex I, complex II, complex IV and pH. *p<0.05; ***p<0.001; ****p<0.0001.

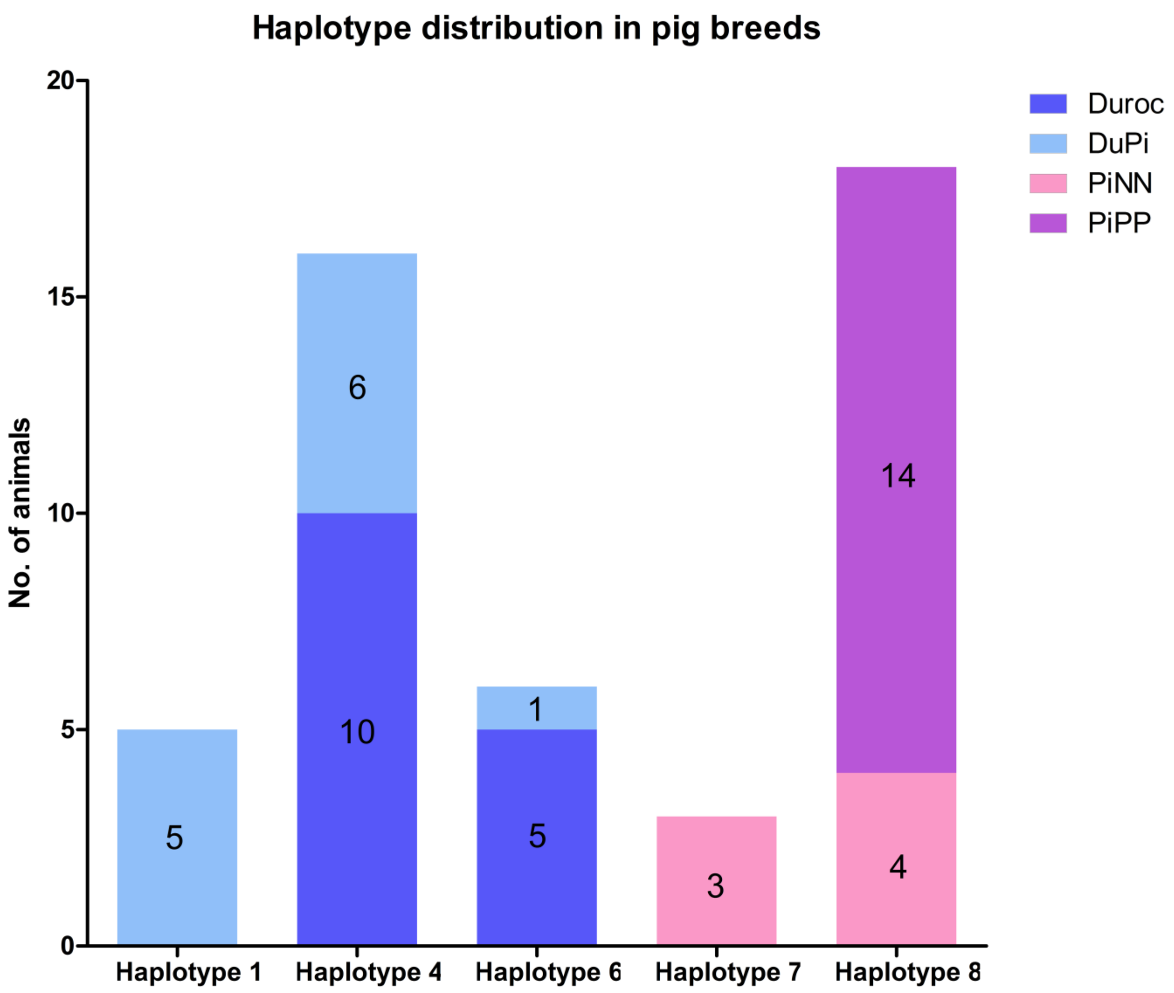

Supplementary Figure 3.

Haplotype distribution in four pig breeds. Blue bar indicates breed Duroc; light blue bar indicates breed DuPi; pink bar indicates breed PiNN; purple bar indicates breed PiPP. The number labelled on each color bar indicates the no. of animals in one particular haplotype.

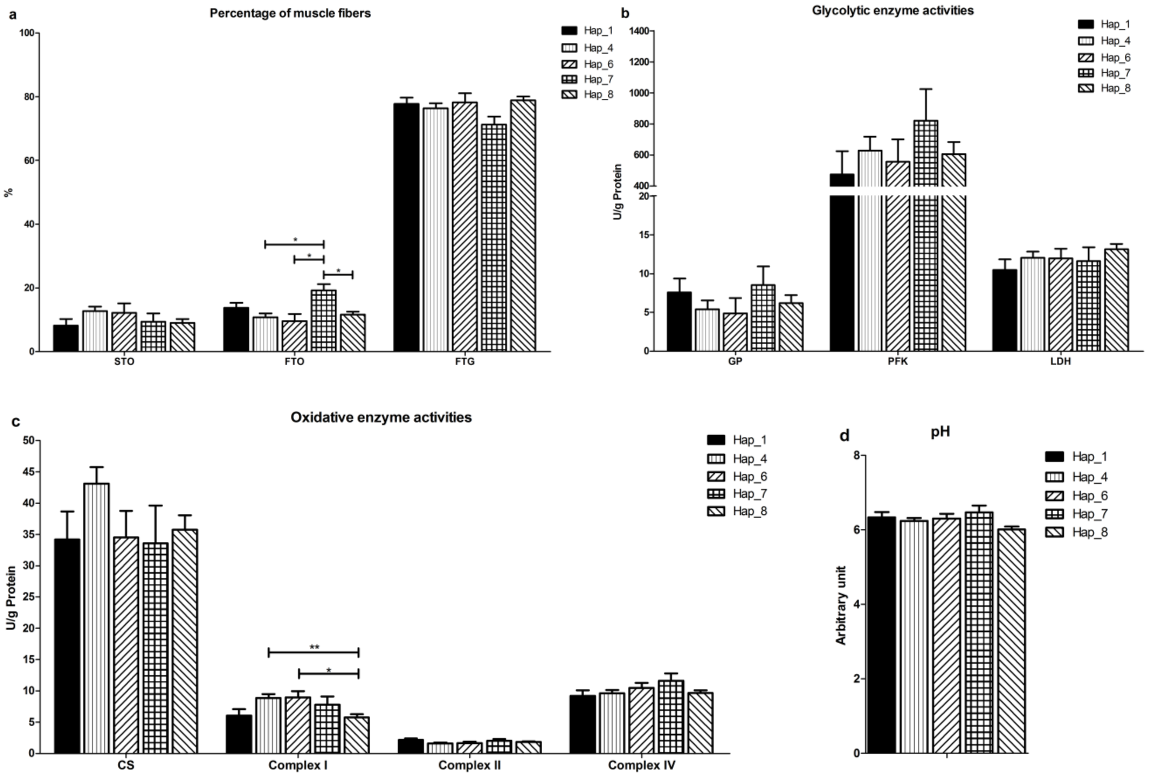

Supplementary Figure 4.

Phenotypes in longissimus muscles of different mitochondrial haplotypes. Least-square means with standard error (Lsmeans ± SE) of (A) percentage of muscle fibers. (B) Glycolytic enzyme activities. (C) Oxidative enzyme activities (D) pH. *p<0.05; **p<0.01.
